# Unique profile of predominant CCR5-tropic in CRF07_BC HIV-1 infections and discovery of an unusual CXCR4-tropic strain

**DOI:** 10.3389/fimmu.2022.911806

**Published:** 2022-09-23

**Authors:** Xiaoyan Hu, Yi Feng, Kang Li, Yueyang Yu, Abdur Rashid, Hui Xing, Yuhua Ruan, Lingling Lu, Min Wei, Yiming Shao

**Affiliations:** ^1^ School of Medicine, Nankai University, Tianjin, China; ^2^ State Key Laboratory for Infectious Disease Prevention and Control, National Center for Acquired Immune Deficiency Syndrome/Sexually Transmitted Diseases (AIDS/STD) Control and Prevention, Chinese Center for Disease Control and Prevention, Beijing, China; ^3^ College of Life Sciences, Nankai University, Tianjin, China; ^4^ Nankai University Second People’s Hospital, Nankai University, Tianjin, China

**Keywords:** HIV-1, CRF07_BC, CXCR4, CCR5, tropism

## Abstract

CRF07_BC is one of the most prevalent HIV-1 strains in China, which contributes over one-third of the virus transmissions in the country. In general, CRF07_BC is associated with slower disease progression, while the underlying mechanisms remain unclear. Our study focused on envelope proteins (Env) and its V3 loop which determine viral binding to co-receptors during infection of cells. We studied a large dataset of 3,937 *env* sequences in China and found that CRF07_BC had a unique profile of predominantly single CCR5 tropism compared with CCR5 and CXCR4 dual tropisms in other HIV-1 subtypes. The percentages of the CXCR4-tropic virus in B (3.7%) and CRF01_AE (10.4%) infection are much higher than that of CRF07_BC (0.1%), which is supported by median false-positive rates (FPRs) of 69.8%, 25.5%, and 13.4% for CRF07_BC, B, and CRF01_AE respectively, with a cutoff FPR for CXCR4-tropic at 2%. In this study, we identified the first pure CXCR4-tropic virus from one CRF07_BC-infected patient with an extremely low CD4^+^T cell count (7 cells/mm^3^). Structural analysis found that the V3 region of this virus has the characteristic 7T and 25R and a substitution of conserved “GPGQ” crown motif for “GPGH”. This study provided compelling evidence that CRF07_BC has the ability to evolve into CXCR4 strains. Our study also lay down the groundwork for studies on tropism switch, which were commonly done for other HIV-1 subtypes, for the long-delayed CRF07_BC.

## Introduction

Human immunodeficiency virus type 1 (HIV-1) remains a significant global public health challenge, with approximately 38.4 million people infected worldwide by 2021 (https://www.unaids.org/en). HIV-1 is one of the most genetically diverse viruses due to its high-mutation and recombination rates, rapid replication rate, and large population size. HIV-1 is represented by four main groups and further subcategorized into nine distinct subtypes and numerous circulating recombinant forms (CRFs) (1, 2). In China, the main epidemic strains of HIV-1 are CRF07_BC, CRF01_AE, and subtype B. CRF07_BC has been the most prevalent strain since it emerged in intravenous drug users (IDUs) in southwest China in 1990s. The virus kept its momentum by spreading rapidly in most parts of the country (3–6). Its high transmission capacity poses great challenges to epidemic prevention and control.

In contrast, in disease progression, CRF07_BC is less pathogenic than CRF01_AE and subtype B (7). Lin’s study showed that individuals infected with CRF07_BC had slower immunological progression (defined as consecutive CD4^+^T cell count <350 cells/mm^3^ more than 3 months after HIV infection was diagnosed) than subtype B (8). Meanwhile, CRF07_BC-infected patients are associated with higher CD4^+^T cell counts and shorter time to achieve immune recovery during combined anti-retroviral therapy (cART) (9). In summary, CRF07_BC has a higher transmission efficiency and slower disease progression. This may be related to high-frequency CCR5 tropism viruses in CRF07_BC, since primary HIV-1 infection is always caused by CCR5 tropism virus (10, 11). HIV-1 utilizes the CD4 receptor and CCR5 co-receptor (C–C chemokine receptor 5, mediating entry of R5-tropic viruses) or the CXCR4 co-receptor (C–X–C chemokine receptor 4, mediating entry of X4-tropic viruses), or both coreceptors to enter host cells (12). Generally, X4−tropic viruses are strongly associated with accelerated disease progression and jeopardizes R5−based HIV−1 cure strategies (13). During subtype B infection, viral variants have acquired the ability to use CXCR4 instead of CCR5 in 40% to 50% of subjects and accelerate the rate of disease progression (14, 15). CRF01_AE exhibits a higher fraction of X4-tropic viruses and faster HIV disease progression compared to non-CRF01_AE viruses (16–18). A rapid outgrowth of a preexisting X4-tropic strain population has been shown in the presence of maraviroc therapy (19). Verheyen’s study has shown a rapid replacement of R5-tropic viruses by a preexisting X4 minority variant in the patient who received an allogeneic transplant with stem cells lacking expression of the CCR5 co-receptor (CCR5Δ32) (20). Therefore, tropism determination is of great significance in the ongoing development of HIV-1 cure strategies.

To our knowledge, until now there is no CRF07_BC strain that can use the CXCR4 co-receptor, except Chen’s study (21). In this study, we systematically analyzed the genotypic and phenotypic characteristics of CRF07_BC strains. We identified the first pure X4-tropic virus from one CRF07_BC-infected patient with an extremely low CD4^+^T cell count (7 cells/mm^3^) and analyzed its characteristic region. This study provides strong evidence that CRF07_BC viruses can utilize the CXCR4 co-receptor and provide valuable experimental materials for the study of the tropism switch mechanism of CRF07_BC viruses.

## Materials and methods

### Collection of samples

In this study, HIV-1 patients’ information and plasma samples were collected from the National HIV-1 Molecular Epidemiological Survey (NHMES). All samples were obtained from newly diagnosed HIV-positive individuals. Moreover, the survey was conducted in a cross-sectional method using stratified random sampling by province. The study was approved by the Ethical Committee of the National Center for AIDS/STD Control and Prevention (ethical review number: X140617334). Each study participant provided written informed consent.

### Viral RNA extraction, cDNA synthesis, and sequence amplification

Viral RNA was extracted from the plasma samples using the QIAamp Viral RNA Mini Kit (Qiagen, Hilden, Germany), and the first-strand cDNA was immediately synthesized by SuperScript III First-Strand Synthesis System (Invitrogen, Carlsbad, USA) according to the manufacturer’s instructions.

HIV-1 *pol* (HXB2:2243-3326) and *env* (HXB2:7002-7663) genes were amplified using nested PCR. The primers are listed in **Table S1**. The PCR conditions are shown in **Table S2**. The HIV-1 *env* (HXB2:7002-7663) gene was amplified using the single genome amplification (SGA) method to detect the minor HIV-1 variant populations. The synthesized cDNA was endpoint diluted in 96-well plates such that fewer than 29 PCRs yielded an amplification product. According to a Poisson distribution, the cDNA dilution that yields PCR products in no more than 30% of wells contains one amplifiable cDNA template per positive PCR more than 80% of the time (22).

The PCR amplified products were directly sequenced by Sangon Biotech (https://www.sangon.com/), and individual sequences were assembled and edited by Sequencher software v4.7.

### Sequence analysis and co-receptor usage prediction

To analyze the phylogenetic relationship among genotypes and clusters, a maximum likelihood phylogenetic tree was constructed using FastTree 2.3 software, with a GTR nucleotide substitution model. The overall mean distance is the arithmetic mean of all individual pairwise distances between taxa. They were calculated by MEGA 11.0.10 using the Poisson model. The online Highlighter tool of the HIV database was used to analyze the nucleotide in a query sequence that matches or does not match with the master sequence. The nucleotide that does not match with the master sequence was assigned a color. The consensus nucleotide sequence, as the master sequence, was made at https://www.hiv.lanl.gov/content/sequence/CONSENSUS/consensus.html. A Highlighter plot was drawn at https://www.hiv.lanl.gov/content/sequence/HIGHLIGHT/highlighter_top.html (23). The sequences containing the V3 region were submitted to the Geno2Pheno_[coreceptor]_ algorithm (https://coreceptor.geno2pheno.org) to predict the co-receptor usage. The results are given with a quantitative value, the false-positive rate (FPR), that defines the probability of falsely classifying an R5 variant as an X4 variant (24).

### Cloning full-length *env* gene from plasma viral RNA

To clone the *env* gene from the extracted plasma viral RNA, cDNA was first synthesized using the SuperScript III First-Strand Synthesis System for RT-PCR with primers 07Rev8 and 1.R3.B3R (**Table S1**). The full-length *env* gene was then amplified using primers HZBOB and HZBCOE for the first-round PCR, and primers HZBIB and HZBCIE for the second-round PCR. Full-length *env* nucleotide sequences were cloned into pcDNA3.1-TOPO-V5-His vector (Invitrogen, Carlsbad, USA) according to the manufacturer’s instructions. The plasmids were analyzed by restriction analysis (*Hin*d III and *Xba*I) to confirm the presence of the insert.

### Pseudovirus production, titration, and coreceptor usage determination

To produce pseudoviruses, 1 μg of envelope expression plasmid and 2 μg of backbone plasmid pSG3Δenv were co-transfected into 4 × 10^5^ 293T cells using PEI-infection transfection reagent and incubated at 37°C with 5% CO_2_ for 48 h. Cell-free supernatants were collected 48 h after transfection and filtered through 0.45 μm filters and stored at -80°C.

The TCID_50_ (Median Tissue Culture Infectious Dose) titer was evaluated using TZM-bl cells. TZM-bl cells (1 × 10^4^) per well were seeded in a 96-well plate. Serial dilutions of virus stocks were added in a final volume of 200 ml/well containing 10 μg/ml of DEAE-dextran. After 48 h postinfection, the cells were lysed for the measurement of luciferase activity.

Viral coreceptor utilization was tested on Ghost cells that stably express CD4 and the coreceptor, either CCR5 or CXCR4. These cells contain the gene of the green fluorescence protein (GFP) driven by the HIV-2 LTR. Upon transfection, viral entry is followed by Tat activation of transcription and GFP becomes expressed (25). Briefly, Ghost cells were seeded in a 24-well plate at the density of 6 × 10^4^ cells/well, On the following day, the same amount of viruses in the presence of 10 μg/ml polybrene was used to infect Ghost cells. After 48 h, infected cells were detected under a fluorescence microscope or harvested and analyzed with BD FACSCalibur Flow Cytometer, and a total of ~15,000 events were scored. Cells without HIV-1 infection were negative control, and cells infected with 1109F (26) and XJ13 (27) which were validated in previous studies were positive controls for CXCR4-tropic and CCR5-tropic, respectively. A fold of 2.5 was set in GFP fluorescence of uninfected cells as positive. To further confirm the coreceptor usage of the viruses, CCR5 antagonist Maraviroc and CXCR4 antagonist AMD3100 were used to treat Ghost cells 1 h prior of infection at the final concentration of 2 nM and 2 μM.

### Sequence alignment and structural modeling

Amino acid sequence identity/similarity was calculated using BioEdit v7.0.9 pairwise alignment for two sequences. A sequence alignment diagram was made at https://espript.ibcp.fr/ESPript/cgi-bin/ESPript.cgi. The crystal structure of envelope glycoprotein gp160 (Protein Data Bank code: 7n6u) was used to model the full-length Env amino acid of In1808-3 and In1808-4. The V3-CXCR4 (28) docking model was used to analyze the interaction between the V3 loop and coreceptor CXCR4. PyMOL 2.3.2 software was used to show the structural diagram.

### Statistical analysis

Statistical analyses and graphs were performed with SPSS 20.0 and GraphPad Prism 8.2.263. The non-parametric Kruskal–Wallis test was applied to compare FPR and CD4^+^T cell counts between groups. To compare the proportion of FPR below 2%, Pearson chi-square (χ^2^) was employed. *P* < 0.05 was considered as statistically significant. Moreover, the significance level for multiple comparisons was calculated by Bonferroni correction.

## Results

### Almost all prevalence of CCR5-tropic viruses in CRF07_BC

**Figure 1:**
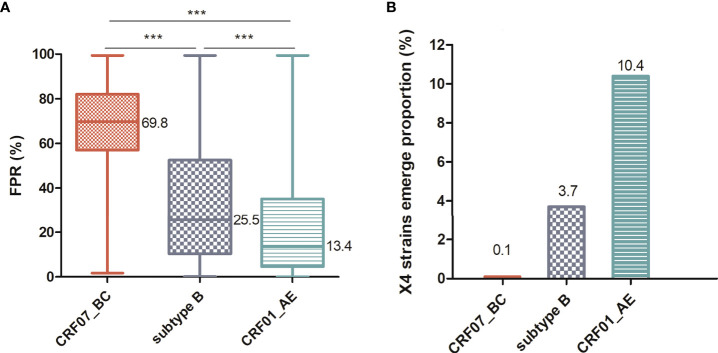
Comparison of the prevalence of X4-tropic strains among different HIV-1 subtypes. **(A)** Comparison of FPR among CRF07_BC (n = 1,123), CRF01_AE (n = 1,235), and subtype B (n = 134) infections. **(B)** X4-tropic strains show a proportion (set FPR<2%) in different HIV-1 subtypes. The middle bars indicate median, box indicates interquartile range, and whiskers indicate minimum and maximum. The statistical difference in FPR was calculated using the non-parametric Kruskal–Wallis test. The percentage of FPR below 2% was compared using the Pearson chi-square (χ^2^) test. *** means *P* < 0.0001.

To systematically compare the genotypic and phenotypic differences among the prevalent HIV-1 strains in China, we conducted a large cross-sectional study using the NHMES dataset. We collected all samples that had both the *env* sequences and CD4^+^T cell counts at the time of diagnosis. We compared the predicted score FPR of the three major viruses and found that the FPR median of CRF07_BC was significantly higher than that of the other two groups (*P* < 0.0001) (**Figure 1A**). The FPR of CRF07_BC mainly ranged between 57% and 82%, while the FPR of CRF01_AE was 4.5%–35%. A comparison of the predicted results with the phenotypic validation results showed that 2% was a more appropriate cutoff value (29). Therefore, we set the cutoff value at 2%, which means that a value below 2% is predictive for an X4 virus whereas a value above 2% reflects an R5 virus. Then, we calculated the proportion of X4 variants in CRF07_BC, CRF01_AE and subtype B. As shown in **Figure 1B**, the proportion of X4 strains was only 0.1% in CRF07_BC, much lower than others.

The frequency distribution of FPR also showed that CRF07_BC had a higher FPR (**Table 1A**). In order to explore the FPR frequency distribution of CRF07_BC more systematically, we further collected 2,814 CRF07_BC partial *env* sequences (one sequence/patient) from the LANL database. A total of 3,937 CRF07_BC *env* sequences from 1996 to 2019 were analyzed. Overall, CRF07_BC still showed a lower fraction of viruses with a low FPR (**Table 1B**). This suggests that CRF07_BC has a unique profile, which is the absolute predominance of the R5-tropic strains. Meanwhile, we can also see from that the lower FPR rate shows a modest increase with time. This indicates that the *env* gene may have a phenotype evolution over time.

**Table 1A:** Analysis of the FPR ratio among different HIV-1 subtypes.

Subtype	<1% ratio^a^(No. of seq)	<2.5% ratio(No. of seq)	<5% ratio(No. of seq)	<10% ratio(No. of seq)	<15% ratio(No. of seq)	<20% ratio(No. of seq)	≥20% ratio(No. of seq)	Total^b^
**CRF07_BC**	0.00% (0)	0.09% (1)	0.62% (7)	2.05% (23)	3.65% (41)	5.25% (59)	94.75% (1,064)	100.00% (1,123)
**Subtype B**	1.49% (2)	3.73% (5)	7.46% (10)	24.63% (33)	35.82% (48)	43.28% (58)	56.72% (76)	100.00% (134)
**CRF01_AE**	2.75% (34)	10.85% (134)	27.69% (342)	45.02% (556)	51.90% (641)	59.92% (740)	40.08% (495)	100.00% (1,235)

**Table 1B:** Analysis of the FPR ratio of CRF07_BC viruses over time.

Time	<1% ratio (No. of seq)	<2.5% ratio (No. of seq)	<5% ratio (No. of seq)	<10% ratio (No. of seq)	<15% ratio (No. of seq)	<20% ratio (No. of seq)	≥20% ratio (No. of seq)	Total
**1996-1999**	0.00% (0)	0.00% (0)	0.33% (1)	1.33% (4)	1.67% (5)	2.00% (6)	98.00% (294)	100.00% (300)
**2000-2009**	0.00% (0)	0.17% (3)	0.57% (10)	1.54% (27)	3.02% (53)	4.45% (78)	95.55% (1,675)	100.00% (1,753)
**2010-2019**	0.11% (2)	0.27% (5)	0.74% (14)	1.96% (37)	3.40% (64)	5.15% (97)	94.85% (1,787)	100.00% (1,884)
**Total**	0.05% (2)	0.20% (8)	0.64% (25)	1.73% (68)	3.10% (122)	4.6% (181)	95.4% (3,756)	100.00% (3,937^c^)

^a^<1% ratio” means the ratio of FPR<1%, “No. of seq” means the number of sequences.

^b^All sequences were collected from the NHMES database.

^c^A total of 3,937 CRF07_BC sequences were collected from the NHMES database (1,123) and LANL database (2,814).

### Individuals infected with X4-tropic viruses displayed a lower CD4^+^T cell count than individuals infected with R5-tropic viruses

**Figure 2:**
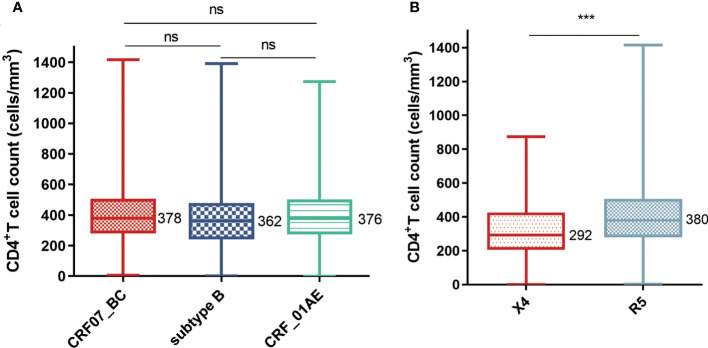
Comparison of the CD4^+^T cell counts **(A)** Comparison of CD4^+^T cell counts among different HIV-1 subtypes (the number of samples of CRF07_BC, subtype B, and CRF01_AE are 1,123, 134, and 1,235). **(B)** Comparison of CD4^+^T cell counts between X4-tropic (n = 135) and R5-tropic (n = 2,357) strain-infected individuals. The middle bars indicate median, box indicates interquartile range, and whiskers indicate minimum and maximum. The statistical difference in CD4^+^T cell count was calculated using the non-parametric Kruskal–Wallis test. *** means *P* < 0.0001. ns, no significance.

To further analyze the characteristics of individuals infected with CRF07_BC, we compared the CD4^+^T cell counts among different subtypes. As shown in **Figure 2A**, the CD4^+^T cell counts of CRF07_BC were higher than those of the other two subtypes but have no significant differences in this dataset. However, when we divided viruses into X4 and R5 groups based on the Geno2Pheno-predicted results and set up the cutoff value at 2%, the CD4^+^T cell counts in the X4 group were significantly lower than those in the R5 group (**Figure 2B**). A similar result has been observed in another study. The X4-tropic virus has the effect of decreasing the CD4^+^T cell count and increasing the risk of clinical disease (30).

### Characteristic analysis of CRF07_BC-infected individuals with low CD4^+^T cell counts

The above results clearly show that CRF07_BC viruses have the predominant R5-tropic, without a shift to X4-tropic in the later stage of infection like the other HIV-1 subtypes. We have followed CRF07_BC-infected individuals for many years and failed to find a single X4-tropic virus. Since the X4 virus is more cytopathic with a low CD4^+^T cell count, we designed a study to fish out the X4-tropic virus in CRF07_BC-infected people with very low CD4^+^T cell counts. We used *pol* and *env* regions to determine correct subtypes and clusters for CRF07_BC viruses (**Figure S1**), since one cannot distinguish further recombinant strains based on only one region. According to the *env* region, not all CRF07_BC strains (determined by *pol*) were suspected to this clade; there were five CRF01_AE strains. This indicates a certain proportion of recombination between CRF07_BC and CRF01_AE and made the *env* region obtain a sequence characteristic of being more favorable to X4 variants. Among these strains, the FPR of the CRF07_BC clade (the FPR median = 69.8%) was significantly higher than that of the CRF01_AE (the FPR median = 3.5%), but we successfully obtained CRF07_BC X4 variants at the predicted level (subject 1809, FPR = 1.7%) (**Table S3**).

### Genotypic and phenotypic analyses of CRF07_BC pseudoviruses

**Figure 3:**
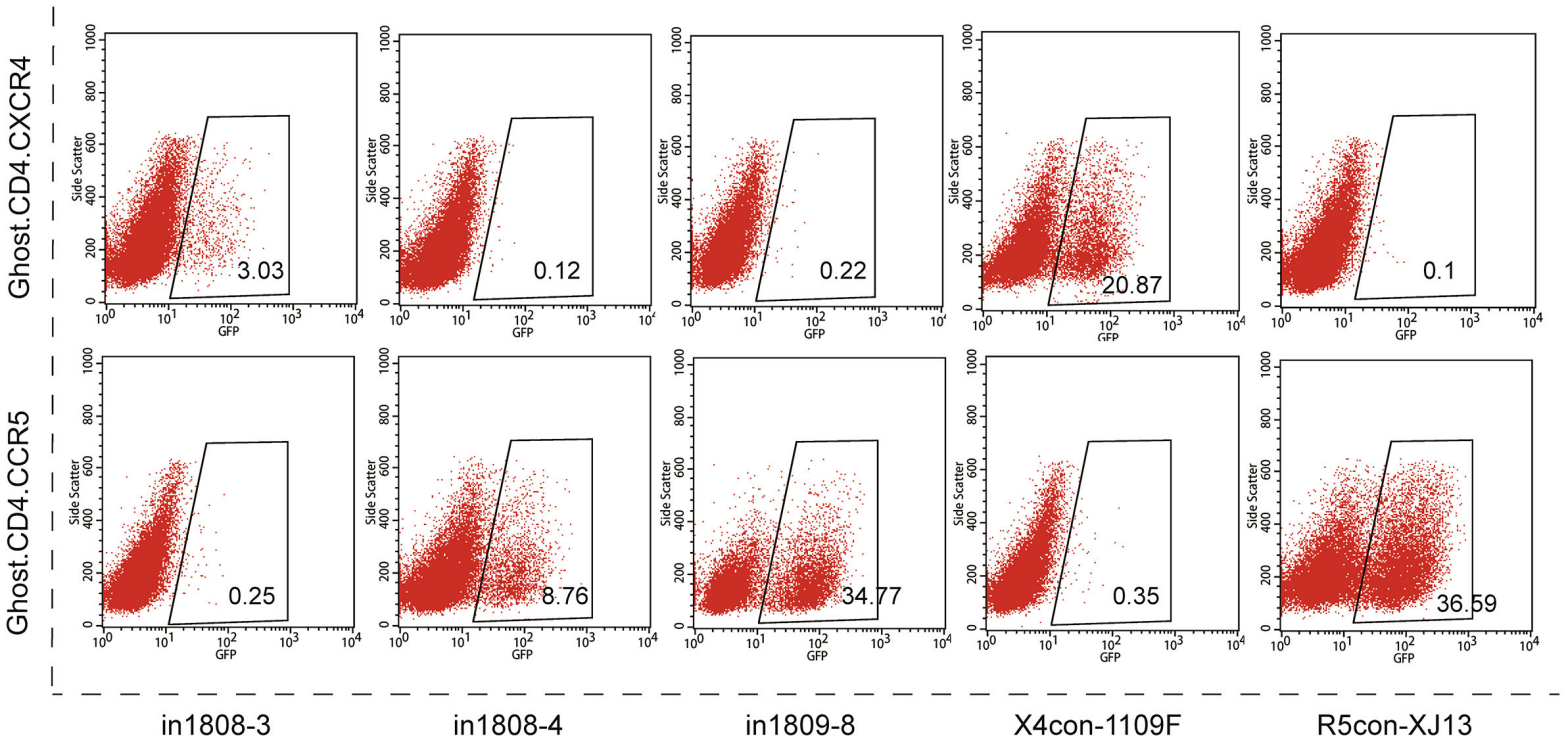
Co-receptor usage of pseudoviruses from individuals 1808 and 1809. Expression of GFP in Ghost.CD4.CXCR4 cells or in Ghost.CD4.CCR5 cells after pseudoviruses infection. Only pseudoviruses In1808-3 can use co-receptor CXCR4. X4con-1109F and R5con-XJ13 as positive control for X4-tropic and R5-tropic, respectively.

We then constructed *env* molecular clones for phenotypic identification. Plasma samples were used to amplify and clone the full-length *env* into the expression vector pcDNA3.1-TOPO-V5-His (**Figure S2**). The viral titers are shown in **Table S5**. The co-receptor usage of pseudoviruses was identified based on the ability to express the GFP reporter in Ghost.CD4.CCR5 and Ghost.CD4.CXCR4. After excluding non-functional and non-CRF07_BC pseudoviruses, 21 molecular clones were analyzed (GenBank: ON157025-ON157045). As shown in **Table 2**, among the variable regions V1–V5, the V1 region has the largest overall mean distance, followed by V5, V4, and V2, while the V3 loop, as the key region for determining tropism, is the most conservative. Of all the pseudoviruses, only In1808-3 showed the ability to use the CXCR4 co-receptor. As shown in **Figure 3**, the GFP expression rate of pseudovirus-infected Ghost.CD4.CXCR4 cells was 3.03%. The pseudoviruses In1808-4 from the same subject showed R5 tropism, while pseudoviruses In1809-8, which were predicted to be X4 strains (FPR = 1.7%), cannot infect Ghost.CD4.CXCR4 cells. Moreover, pseudoviruses could be well inhibited by corresponding antagonists AMD3100 and Maraviroc (**Figure S3**). Genetic analysis revealed that compared to the consensus sequence, the only X4 pseudoviruses In1808-3 showed greater variation in the V3 region with a higher V3 charge and had a longer V2 region and a shorter V4 region (**Table 2**). Mild’s research showed the same results; the V2 region was significantly longer in viruses of CCR5-to-CXCR4 switch populations (SP) compared to viruses of nSP, and the V3 charge increased with time in R5 populations from SP (31).

**Table 2:** Genotypic and phenotypic analyses of full-length envelope.

Pseudoviruses^a^	V1^b^	V2^b^	V3^b^	V4^b^	V5^b^	V3 loop^d^	FPR(%)	V3 net charge	Coreceptor^f^
	1.29^c^	0.39^c^	0.14^c^	0.54^c^	0.71^c^	CTRPGNNTRKSIRIGPGQTFYATGDIIGDIRQAHC^e^			
In1808-3	23	47	35	25	12	————n—t————–—–———**h**avf——er––——–—k———	0.8	7	X4
In1808-4	23	41	35	25	11	————n——————–—–———**h**——f————t––——–—k———	88.5	5	R5
In1809-4	19	41	35	36	12	——————————g–g—–————————rea––——–—————	1.7	4	R5
In1809-8	19	39	35	36	12	——————————g–g—–————————rea––——–—————	1.7	4	R5
In1810-1	23	44	35	36	12	————n——————–—–———————————––—n—–—————	86.5	5	R5
In1810-3	22	44	35	30	12	a———n——————–—–———————————––—n—–—————	53.8	5	R5
In1813-1	25	39	35	33	12	————n——————–—–———————————––—–—–—————	83	4	R5
In1813-8	19	39	35	33	12	————n——————–—–———————————––—–—–—————	83	4	R5
In1821-1	17	41	35	26	12	———————————–—–——————————ev–—–—–—————	45.4	4	R5
In1821-8	19	41	35	31	12	———————————–—–——————————q–—–—–—————	53.5	5	R5
In1821-9	19	41	35	31	12	———————————–—–——————————q–—–—–—————	53.5	5	R5
In1822-8	20	40	34	26	12	—i——–———————–—–———————————––——–————y—	42.3	3	R5
In1825-4	22	44	35	30	12	———–———————–—–———————————––——–——————	69.8	4	R5
In1837-2	18	45	35	28	12	—i——n——r—————–—–———a————————––——–———y—	13.8	4	R5
In1837-3	19	49	35	28	12	—i——n——r—————–—–———a————————––——–———y—	13.8	4	R5
In1838-1	9	44	35	28	12	—–——–——–—————–—–———a————————––——–———–—	34.3	4	R5
In1838-3	9	44	35	28	12	—–——–——–—————–—–———a————————––——–———–—	34.3	4	R5
In1839-4	22	44	35	30	12	———–———————–—–———————————––——–——————	69.8	4	R5
In1841-9	22	44	35	30	12	———n———————–—–————m————e—––——–——————	69.8	4	R5
In1846-7	19	37	35	33	12	———–———————–—–———————————––——–——————	56.9	4	R5
In1852-8	23	38	35	32	12	—i——n——r—————–—–———a—————m——––——–———y—	63.1	2	R5

^a^Pseudoviruses were named on the principle of “individual serial number-clone serial number”. For example, In1808-3 means number #3 clone of individual 1808.

^b^Number of amino acids in variable regions V1–V5.

^c^Overall mean distance of the V1–V5 amino acid sequences, calculated by MEGA 11.0.10.

^d^Amino acid sequences compared to consensus sequences; dashes denote sequence identity, and dots denote absence of amino acids.

^e^The consensus sequences of all pseudoviruses’ V3 region with crown motif in bold.

^f^The coreceptor usage is the result from phenotypic testing.

### Sequence and structural analyses of R5 and X4 envelope protein from subject 1808

**Figure 4:**
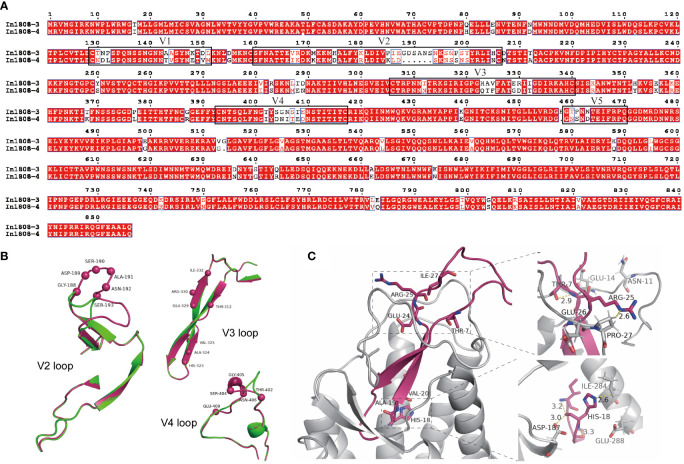
Sequence and structural analyses of R5 and X4 Envs from subject 1808. **(A)** Full-length Env amino acid alignment of pseudoviruses In1808-3 (X4) and In1808-4 (R5). Variable regions V1–V5 are shown in boxes. **(B)** The structural alignment of V2 loop, V3 loop, and V4 loop; the whole structure is shown in cartoon, the main difference amino acids are highlighted by spheres model, and In1808-3 in warm pink, In1808-4 in green. **(C)** Structural analysis for the V3 region features points in binding of co-receptors CXCR4 using the V3-docking model. The whole structure is shown in cartoon, and the key residues are shown in sticks.

As mentioned above, we successfully constructed a X4-tropic CRF07_BC pseudovirus from subject 1808. The amino acid sequence analysis showed that the identity/similarity for In1808-3 and In1808-4 was 0.8961494/0.8949825. Then, the different area is the key region that determines the tropism. As shown in **Figure 4A**, there was a considerable variation at the V2 and V4 regions, with six amino acids inserted at the V2 region in In1808-3 (X4-tropic). In the V3 region, a seven-amino acid variation was also found that was thought to be a key determinant of tropism. Some of these altered amino acids are consistent with the characteristic of the previously reported X4 strains. For example, 11/25 and net charge rules (32–34) had an amino acid substitution of the highly conserved “GPGQ” crown motif for “GPGH”.

We next used homology models for the structural alignment of the full-length Env. **Figure 4B** shows the structural differences of key variation regions in V2, V3, and V4 of In1808-3 and In1808-4. The alignment result showed that there was a significant structural difference at V2 and V4 regions. Due to the insertion of six amino acids into In1808-3 V2, its main chain structure was greatly changed. Similarly, in the 402-409 region of V4, the main chain structure also changed, while there was no significant difference in the main chain structure of the V3 region. **Table 2** shows that the V3 loop of In1808-3 has characteristic amino acids 7T, 18H, and 25R compared with consensus sequences and other sequences. Then, a V3-CXCR4 (28) docking model was used to analyze the interaction between In1808-3 V3 and co-receptor CXCR4. As shown in **Figure 4C**, the V3 loop is inserted into CXCR4 and the variable sites were mainly located at the tip of V3 and in the region that interacts with the binding pocket tip. The upper right of **Figure 4C** showed key amino acids THR-7 and ARG-25. THR-7 interacts with V3 loop THR-8, and ARG-25 interacts with PRO-27 of co-receptor CXCR4. As shown, the CXCR4-binding pocket around ARG-25 contains negatively charged amino acids, which may favor for interacting with positively charged amino acids ARG-25. The lower right area of **Figure 4C** shows the characteristic amino acid HIS-18, which interacts with the co-receptors ILE-284 and ASP-187 and with the V3 loop ILE-13 and PRO-16. Meanwhile, the acidic environment at the binding pocket may also be more suitable for the alkaline amino acid HIS-18.

### SGA to detect the HIV-1 variant populations with different co-receptor usage in HIV-1-infected plasma

**Figure 5:**
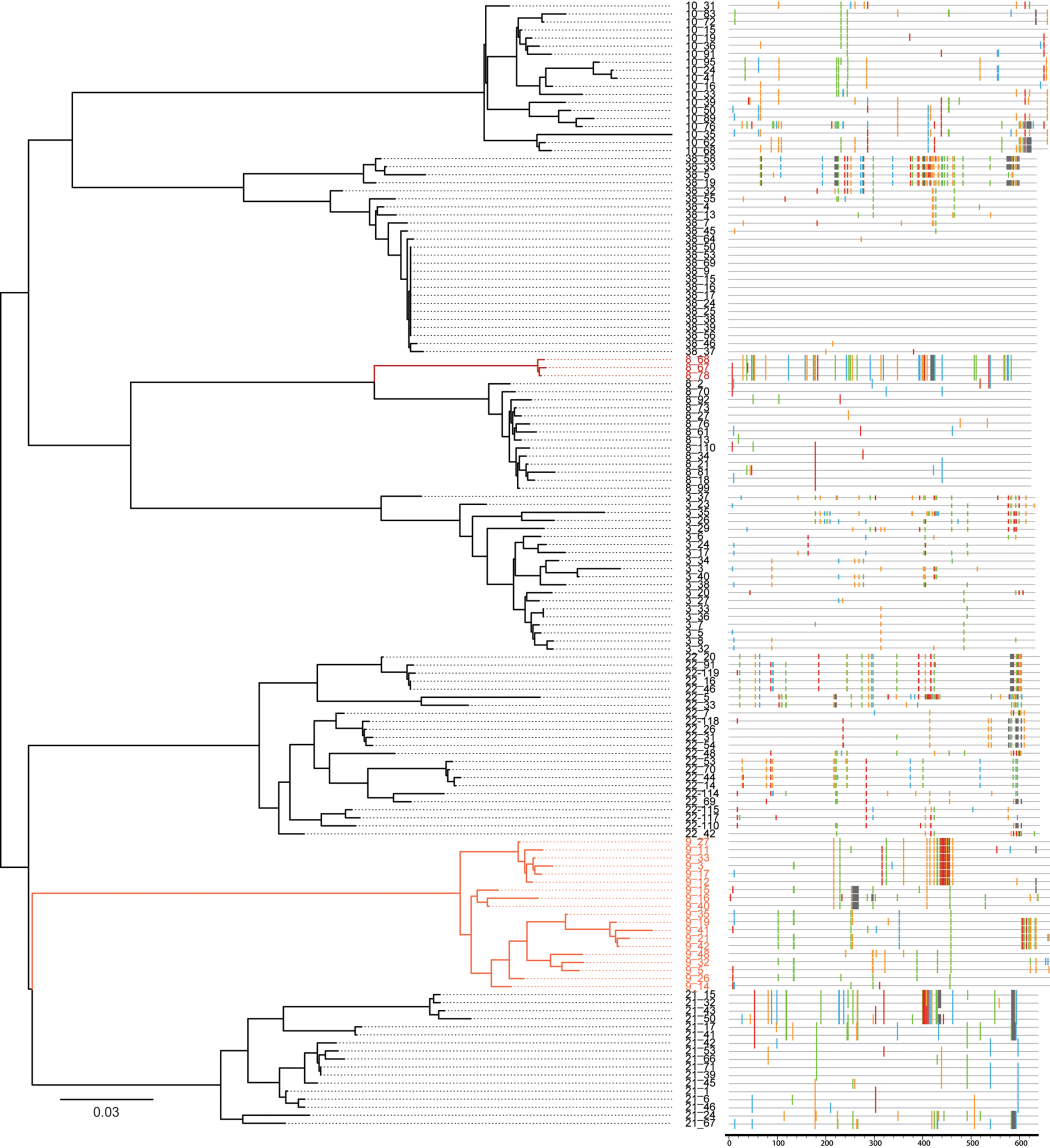
SGA to detect the HIV-1 variant populations with different coreceptor usages. SGA-derived *env* sequences from seven HIV-1-infected plasma samples were examined by phylogenetic tree construction (left panels) and Highlighter plot analysis (right panels). Sequences predicted to be X4-tropic are in red face, and the darker the color, the lower the FPR (0.8% and 1.7%). The corresponding Highlighter diagrams denote the locations of nucleotide sequence substitutions in each *env* sequence in comparison to their consensus sequence (the consensus sequence for each individual is not shown). Nucleotide substitutions and gaps are color coded (A, green; C, blue; G, orange; T, red; and GAP, gray). In individual 1808, both R5-tropic (FPR = 88.5%) and X4-tropic (FPR = 0.8%, in deep red) strains were present and their sequences were quite different. All the sequences are predicted to be X4-tropic (FPR = 1.7%, in light red) in individual 1809.

Considering that we obtained both X4-tropic and R5-tropic viruses from the same sample 1808, we then explored whether this phenomenon also existed in other samples and the distribution of X4 and R5 viruses. Therefore, we used the SGA method to detect the distribution of viral quasispecies in the same plasma samples to find the minor HIV-1 variant populations with the different coreceptor usage. One hundred forty SGA-derived sequences were analyzed using the maximum likelihood phylogenetic tree method together with the Highlighter a sequence visualization tool. Phylogenetic analysis showed that sequences from the same sample clustered together (**Figure 5** left). Highlighter plots depicted the positions and identities of nucleotide polymorphisms, insertions, and deletions of each sequence compared to their consensus sequence (**Figure 5** right). Each sample showed distinct lineages in the phylogenetic tree and different degrees of variation in the Highlighter analysis. Then, we further analyzed the V3 regions of these SGA-derived sequences. As shown in **Table S4**, all subjects have at least two different amino acid sequences in the V3 region, and subject 1821 had five different types of V3 regions. Although there were only two different V3 regions in individual 1808, the FPR varied widely (88.5% and 0.8%), leading to different co-receptor usage. Zhou’s study also showed that non-X4 lineages were not skewed toward lower FPR scores in X4-containing populations (35). This result was also consistent with the pseudovirus result. As shown in the phylogenetic tree, in subject 1808, the R5 and X4 (in deep red) strains are mapped with distinct clusters. X4 variants were not detected by conventional Sanger sequencing and were the minor population in the plasma (17.6%). Analogously, the four of the seven individuals, which are subject 1803, 1808, 1810, and 1838, had a minor population with a lower FPR. This may be one of the reasons why X4 variants are so challenging to be detected, even though CRF07_BC can break through the higher genetic barrier to acquire the ability to use CXCR4 co-receptors; these X4 strains are still in a minor population.

## Discussion

CRF07_BC has become the most prevalent strain (39.7%) in China and plays an important role in interprovincial transmission (6). In general, CRF07_BC was associated with slower disease progression, while the underlying mechanisms remain unclear. Some studies suggest that the emergence of X4 variants is accompanied by rapid disease progression (14, 36, 37). In subtype B and CRF01_AE, many X4 variants have been reported. One of the discrepancies was the low frequency of CXCR4 usage among subtype C (38). In the early phenotypic studies, all the isolated subtype C strains use the CCR5 co-receptor (39, 40). A later study from South Africa has reported a higher prevalence of X4 variants among subtype C (41). However, no or very few X4-tropic viruses have been found in CRF07_BC. In this work, we collected and analyzed a large CRF07_BC *env* sequence dataset from 1996 to 2019. We identified the characteristics of CRF07_BC, namely, the absolute predominance of the R5-tropic strains. Our results showed that the predicted ratio of X4 tropic viruses was only 0.1% in CRF07_BC while 10.4% in the CRF01_AE (set FPR<2%). Li’s study also showed a higher prevalence of X4 viruses among CRF01_AE in China (42). Maeda’s (43) study showed that in the phenotypic assay, the frequency of X4 variants was 16.1%. Taken together, the frequency of X4 variants in CRF01_AE is high, and the FPR set as 2% in this clade is a relatively accurate and strict cutoff. While in our study, phenotypic verification of CRF07_BC showed the only X4 variants with an FPR value of 0.8%, the subtype C X4 variants 1109F with FPR of 1.7%. Therefore, we hypothesized that CRF07_BC may have an even more stringent FPR value for X4-tropic than subtype C and other subtype, since In1809-8 (CRF07_BC, FPR = 1.7%) is still a R5-tropic virus. Moreover, this requires more phenotypic validation results to support this idea. Judicate’s study demonstrated the limitation of currently available genotypic algorithms for predicting co-receptor inference among non-B subtypes (44). Therefore, the phenotypic tropism dataset of non-B subtypes is valuable for retraining the genotypic prediction algorithms to enhance their performance.

A previous study of HIV-1 subtype B infection evidenced 15.9% of patients harboring X4 viruses (45). A sequence predicted a level study of HIV-1 subtype C and showed that 8.2% of early sequences were X4 (set FPR <10%) (46), while our study showed that at Geno2Pheno 10% FPR, the frequency of X4 variants was just 1.73% (**Table 1B**). Although the env region of CRF07_BC is mainly derived from subtype C, there are still differences in the utilization of the co-receptor. We also searched full-length *env* sequences (Env CDS) with co-receptor information (“only CXCR4”) from the LANL database. The results showed that the number of “only CXCR4” sequences is 110, 37, and 17 in subtype B, subtype C, and CRF01_AE, respectively. No “only CXCR4” sequence has been found in CRF07_BC. When searched for “all CXCR4”, we found that a study reported a pseudovirus, generated from a CRF07_BC-infected individual BJOX37, which can use CCR5, CXCR4, and FPRL-1 co-receptors tested on NP-2 cells (21). We obtained the BJOX37 plasmid with a kind offer from the author and tested its infectivity on Ghost.CD4.CCR5 and Ghost.CD4.CXCR4 cells in comparison with the our In1808-3 pseudoviruses. The result showed that the predominant co-receptor usage for BJOX37 is CCR5 (3.27%), although it may also slightly use the CXCR4 co-receptor (0.37%), while In1808-3 predominantly uses the CXCR4 co-receptor (3.77%) and rarely uses the CCR5 co-receptor (0.31%). The major infectivity of BJOX37 and In1808-3 can be blocked by CCR5 inhibitor Maraviroc and CXCR4 inhibitor AMD3100, respectively. By the Geno2Pheno _[Coreceptor]_ algorithm, the FPRs for BJOX37 and In1808-3 are 69.8% and 0.8%, respectively. In addition, the V3 loop amino acid sequences of the BJOX37 did not reveal any unique signatures for the X4-like characteristic, but In1803-3 did at the 7T, 18H, and 25R positions. To our knowledge, there is no well-documented report of the CXCR4-only tropism CRF07_BC virus in the past and In1808-3 may be the first reported CRF07_BC virus using the only CXCR4 co-receptor.

Quasispecies analysis showed that viruses with a high FPR were the dominant viruses in most samples. This phenomenon makes it difficult to detect X4 variants by conventional PCR, even though X4 variants are already present in the plasma samples. Therefore, our study provides strong evidence that the CRF07_BC viruses can evolve into X4 variants, while the low prevalence of X4 variants in CRF07_BC may be due to the following reasons. The transmission efficiency of CRF07_BC X4 variants is low, since the transmission is generally mediated by R5 variants (47, 48). The replication capability of X4 variants may be restricted, due to the increased neutralization sensitivity of X4 variants compared to coexisting R5 strains (49). When we identified CRF07_BC X4 variants, we started focusing on the individual who was infected with them. The X4 variant-infected individual 1808 was diagnosed in April 2018 and died in March 2019. The time duration between diagnosis and death was too short; we only collected the CD4^+^T cell count (7 cells/mm^3^) and CD8^+^T cell count (802 cells/mm^3^) at the time of diagnosis, while the samples were collected before antiviral therapy, so the presence of X4 in the patient was not associated with drug selective pressure. In a further study, we will determine the virus prevalence in the region where the X4 variant-infected individual 1808 lived, to fully understand the tropism switch mechanism at viral and host factor levels.

## Data availability statement

The datasets presented in this study can be found in online repositories. The names of the repository/repositories and accession number(s) can be found below: NCBI, accession IDs: ON157025-ON157045.

## Ethics statement

This study was reviewed and approved by the National Center for AIDS/STD Control and Prevention. The patients/participants provided their written informed consent to participate in this study. Written informed consent was obtained from the individual(s) for the publication of any potentially identifiable images or data included in this article.

## Author contributions

YS, XH, MW, and YF conceived and designed the manuscript. XH performed the experiments and wrote the manuscript. KL, YY, AR, HX, YR, and LL participated in the data analysis. XH, YS, YF, AR, KL and MW revised the manuscript. All authors contributed to the article and approved the submitted version.

## Funding

This work was supported by Projects of International Cooperation from the Ministry of Science and Technology of China (Grant number: 2016YFE0107600), Science Priority Grant from the State Key Laboratory of Infections Disease Prevention and Control (Grant number: 2019SKLID602) and National Science and Technology Major Project of China (grant number: 2018ZX10721102-006).

## Acknowledgments

We thank National Center for AIDS/STD Control and Prevention, China CDC and the School of Medicine, Nankai University for assistance during the study. We are grateful to all the members of National HIV-1 Molecular Epidemiological Survey (NHMES). We are indebted to the HIV-1 infected patients from whom the samples were obtained in the present study. Meanwhile, we would also like to thank the reviewers of this manuscript for their important insights and suggestions during the review process.

## Conflict of interest

The authors declare that the research was conducted in the absence of any commercial or financial relationships that could be construed as a potential conflict of interest.

## Publisher’s note

All claims expressed in this article are solely those of the authors and do not necessarily represent those of their affiliated organizations, or those of the publisher, the editors and the reviewers. Any product that may be evaluated in this article, or claim that may be made by its manufacturer, is not guaranteed or endorsed by the publisher.
