# Supplementary material for: Unique profile of predominant CCR5-tropic in CRF07_BC HIV-1 infections and discovery of an unusual CXCR4-tropic strain
Source: Front Immunol. 2022 Sep 23;13:911806. doi: 10.3389/fimmu.2022.911806 (PMC9540210; doi:10.3389/fimmu.2022.911806)
Supplement: Supplementary file 1 [file DataSheet_1.docx]

Supplementary Material

Table S1. The PCR primers

| **Region** | **Round** | **Primer** | **Primer Sequence** | **Positions of HXB2** |
| --- | --- | --- | --- | --- |
| pol | first | F1a | 5’-TGAARGAITGYACTGARAGRCAGGCTAAT -3’ | 2057-2085 |
|  |  | F1b | 5’- ACTGARAGRCAGGCTAATTTTTTAG -3’ | 2068-2092 |
|  |  | R1 | 5’- ATCCCTGCATAAATCTGACTTGC -3’ | 3370-3348 |
|  | second | F2 | 5’- CTTTARCTTCCCTCARATCACTCT -3’ | 2243-2266 |
|  |  | R2 | 5’- CTTCTGTATGTCATTGACAGTCC -3’ | 3326-3304 |
| env | first | 44F | 5’- ACAGTRCARTGYACACATGG-3’ | 6954–6973 |
|  |  | 35R | 5’- CACTTCTCCAATTGTCCITCA-3’ | 7668–7648 |
|  | second | DR7m4 | 5’-TGTAAAACGACGGCCAGTCTGTTAAATGGYAGYCTAGCTC-3’ | 7002-7021 |
|  |  | DR8m4 | 5’-CAGGAAACAGCTATGACCCTCCAATTGTY CCTCATAT-3’ | 7663-7645 |
|  |  | 07Rev8 | 5’-CCTARTGGGATGTGTACTTCTGAACTT-3’ | 5219-5193 |
|  |  | 1.R3.B3R | 5’-ACTACTTGAAGCACTCAAGGCAAGCTTTATTG-3’ | 9611-9642 |
| full-  lenght env | first | HZBOB | 5’-TAGAGCCTTGGAAGCATCCAGGAAGTCAG-3’ | 5853-5881 |
|  |  | HZBCOE | 5’-TAGCCCTTCCAGTCCCCCCTTTTCTTTTA-3’ | 9096-9068 |
|  | second | HZBIB | 5-CACCGATCAAGCTTTAGGCATCTCCTATGGCAGGAAGAAG-3’ | 5944-5983 |
|  |  | HZBCIE | 5’-GTCTCGAGATACTGCTCCCACCCCAT-3’ | 8904-8879 |

Table S2. The PCR conditions

| **Region** | **Round** | **conditions** |
| --- | --- | --- |
| pol | first | 94℃ for 2 min; 35 cycles of 94℃ for 15 s, 50℃ for 20 s and 72℃ for 2 min; 72℃ for 10 min; 4℃ hold. |
|  | second | 94℃ for 2 min; 30 cycles of 94℃ for 15 s, 55℃ for 20 s and 72℃ for 2 min; 72℃ for 10 min; 4℃ hold. |
| env | first | 94℃ for 2 min; 30 cycles of 94℃ for 15 s, 55℃ for 20 s and 72℃ for 2 min; 72℃ for 10 min; 4℃ hold. |
|  | second | 94℃ for 2 min; 5 cycles of 94℃ for 15 s, 54℃ for 20 s and 72℃ for 1 min; 30 cycles of 94℃ for 15 s, 58.5℃ for 20 s and 72℃ for 1 min;72℃ for 10 min; 4℃ hold. |
| full-  lenght env | first | 98℃ for 2 min; 30 cycles of 98℃ for 10 s, 55℃ for 5 s and 72℃ for 4 min; 72℃ for 10 min; 4℃ hold. |
|  | second | 98℃ for 2 min; 30 cycles of 98℃ for 10 s, 58℃ for 5 s and 72℃ for 4 min; 72℃ for 10 min; 4℃ hold. |

Table S3. Characteristics analysis of infected individuals with low CD4^+^T cell counts

| **Subject** | **Clinical characteristics** | | | | | **Sequence characteristics** | | |
| --- | --- | --- | --- | --- | --- | --- | --- | --- |
|  | Age  (yrs) | Sex^a^ | CD4 counts  (cells/mm^3^) | Infection  route ^b^ | Region | Sub pol^c^ | Sub env | FPR (%) ^d^ |
| 1803 | 51 | M | 29 | HE | Chongqing | 07BC_O | 07_BC | 98.6 |
| 1834 | 32 | M | 46 | HE | Jiangxi | 07BC_O | 07_BC | 95.7 |
| 1835 | 41 | F | 28 | HE | Sichuan | 07BC_O | 07_BC | 89.3 |
| 1808 | 60 | M | 7 | HE | Chongqing | 07BC_O | 07_BC | 88.5 |
| 1810 | 48 | M | 22 | HE | Chongqing | 07BC_N | 07_BC | 86.5 |
| 1813 | 58 | M | 48 | MSM | Chongqing | 07BC_N | 07_BC | 83 |
| 1830 | 61 | M | 32 | HE | Hunan | 07BC_N | 07_BC | 83 |
| 1856 | 36 | F | 43 | HE | Shanxi | 07BC_O | 07_BC | 83 |
| 1823 | 52 | M | 40 | HE | Hubei | 07BC_N | 07_BC | 79.5 |
| 1842 | 66 | M | 34 | HE | Sichuan | 07BC_O | 07_BC | 75.6 |
| 1845 | 25 | M | 41 | MSM | Ningxia | 07BC_N | 07_BC | 74.6 |
| 1820 | 37 | M | 36 | HE | Guangdong | 07BC_O | 07_BC | 70.1 |
| 1825 | 60 | M | 37 | HE | Hebei | 07BC_N | 07_BC | 69.8 |
| 1839 | 45 | M | 37 | HE | Jiangxi | 07BC_N | 07_BC | 69.8 |
| 1840 | 30 | M | 28 | MSM | Liaoning | 07BC_N | 07_BC | 69.8 |
| 1841 | 52 | F | 39 | HE | Sichuan | 07BC_O | 07_BC | 69.8 |
| 1855 | 62 | M | 49 | HE | Shaanxi | 07BC_O | 07_BC | 69.8 |
| 1852 | 54 | M | 30 | MSM | Shaanxi | 07BC_N | 07_BC | 63.1 |
| 1822 | 59 | M | 2 | MSM | Hubei | 07BC_N | 07_BC | 58.3 |
| 1802 | 58 | M | 27 | HE | Chongqing | 07BC_O | 07_BC | 56.9 |
| 1846 | 45 | M | 22 | HE | Qinghai | 07BC_N | 07_BC | 56.9 |
| 1821 | 27 | M | 9 | HE | Guizhou | 07BC_O | 07_BC | 53.5 |
| 1801 | 38 | M | 36 | HE | Chongqing | 07BC_N | 07_BC | 50.5 |
| 1843 | 42 | M | 18 | HE | Sichuan | 07BC_O | 07_BC | 49.3 |
| 1851 | 45 | M | 35 | MSM | Shaanxi | 07BC_N | 07_BC | 49.3 |
| 1819 | 60 | M | 20 | HE | Guangdong | 07BC_O | 07_BC | 48.4 |
| 1831 | 45 | M | 18 | IDU | Jilin | 07BC_O | 07_BC | 44.8 |
| 1814 | 44 | M | 41 | HE | Chongqing | 07BC_N | 07_BC | 42.7 |
| 1804 | 47 | M | 45 | HE | Chongqing | 07BC_O | 07_BC | 37.1 |
| 1838 | 65 | M | 3 | HE | Jiangxi | 07BC_O | 07_BC | 34.3 |
| 1812 | 53 | M | 17 | HE | Chongqing | 07BC_N | 07_BC | 23.9 |
| 1837 | 50 | M | 35 | HE | Jiangxi | 07BC_O | 07_BC | 13.8 |
| 1809 | 54 | F | 9 | HE | Chongqing | 07BC_O | 07_BC | 1.7 |
| 1816 | 42 | M | 48 | MSM | Guangdong | 07BC_N | 01_AE | 1.7 |
| 1826 | 35 | M | 3 | HE | Hainan | 07BC_N | 01_AE | 3.4 |
| 1828 | 48 | M | 40 | MSM | Heilongjiang | 07BC_O | 01_AE | 3.5 |
| 1859 | 44 | F | 36 | HE | Hunan | 07BC_N | 01_AE | 8.1 |
| 1853 | 53 | M | 41 | MSM | Shaanxi | 07BC_N | 01_AE | 77.1 |

^a^ M, male; F, female.

^b^ HE, heterosexual; MSM, men who have sex with men; IDU, intravenous drug use.

^C:^ 07BC_O is the original CRF07_BC, circulating in intravenous drug users and heterosexuals; The 07BC_N is a new cluster, identified mostly in men having sex with men.

^d^ FPR, false-positive rate in Geno2Pheno_[coreceptor]_.

Table S4. Characteristics Analysis of SGA-derived V3 loop

| **Subject** | **V3 loop** | **FPR (%)** | **No. of SGA** |
| --- | --- | --- | --- |
| 1808 | CTRPNNTTRKSIRIGPGHAVFATERIIGDIRKAHC | 0.8 | 3 |
|  | ------n----------qtf---gd-t-------- | 88.5 | 14 |
| 1809 | CTRPGNNTRKGIGIGPGQTFYAREAIIGDIRQAHC | 1.7 | 10 |
|  | ----------n------------------------ | 1.7 | 9 |
| 1803 | CTRPNNNTRKSVRIGPGQTFFATGEIIGDIRQAHC | 90.9 | 2 |
|  | -------------------------------e--- | 96.2 | 1 |
|  | ----------------------------n--e--- | 96.7 | 1 |
|  | ------------------------d---n--e--- | 98.6 | 16 |
| 1810 | CARPNNNTRKSIRIGPGQTFYATGDIIGNIRQAHC | 53.8 | 3 |
|  | -t--------------------------------- | 86.5 | 16 |
| 1821 | CTRPGNNTRKSIRIGPGQTFYATGEVIGDIRQAHC | 45.4 | 4 |
|  | ------------------------qi--------- | 53.5 | 5 |
|  | ----n-------------------q---n------ | 66 | 4 |
|  | ----n-------------------qi--------- | 72 | 2 |
|  | --------k-------------------------- | 73.3 | 2 |
| 1822 | CIRPGNNTRKSIRIGPGQTFYATDIIGDIRQAYC | 42.3 | 16 |
|  | ----s----------------------------- | 65.9 | 5 |
|  | -e--n----------------------------- | 68.6 | 2 |
| 1838 | CTRPGNNTRKSIRIGPGQAFYATGDIIGDTRQAHC | 23.9 | 4 |
|  | -----------------------------i----- | 34.3 | 21 |

Table S5. Pseudovirus titers

| **Pseudovirus** | **Infectious titer (TCID_50_/mL)**  **(TCID_50_/ml)** | **Pseudovirus** | **Infectious titer**  **(TCID_50_/mL)** | **Pseudovirus** | **Infectious titer**  **(TCID_50_/mL)** |
| --- | --- | --- | --- | --- | --- |
| In1808-3 | 559 | In1821-1 | 6,250 | In1838-3 | 559 |
| In1808-4 | 6,250 | In1821-8 | 2,795 | In1839-4 | 13,975 |
| In1809-4 | 69,877 | In1821-9 | 559 | In1841-9 | 6,250 |
| In1809-8 | 69,877 | In1822-8 | 559 | In1846-7 | 13,975 |
| In1810-1 | 69,877 | In1825-4 | 69,877 | In1852-8 | 31,250 |
| In1810-3 | 69,877 | In1837-2 | 13,975 | X4con-1109F | 1,250 |
| In1813-1 | 13,975 | In1837-3 | 2,795 | R5con-XJ13 | 156,250 |
| In1813-8 | 31,250 | In1838-1 | 559 |  |  |


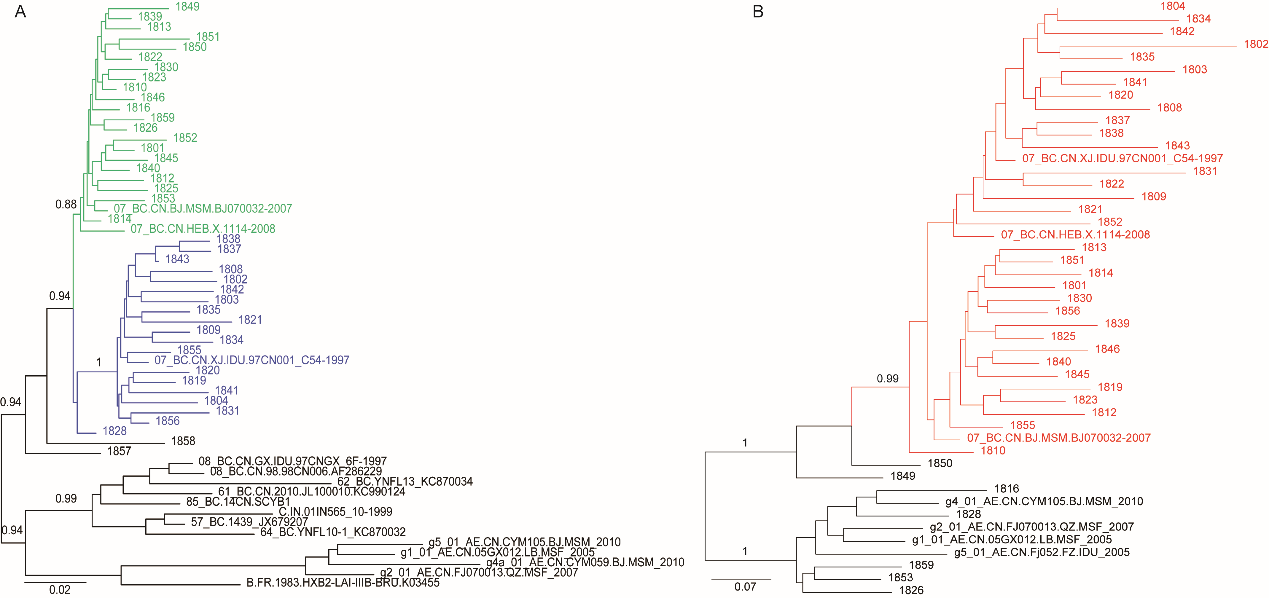


Fig.S1 Phylogenetic analysis of CRF07_BC-infected individuals with low CD4 T cell counts. (A)The phylogenetic tree based on *pol* region and (B) *env* region were constructed using the approximate maximum likelihood method with FastTree, version 2.3.


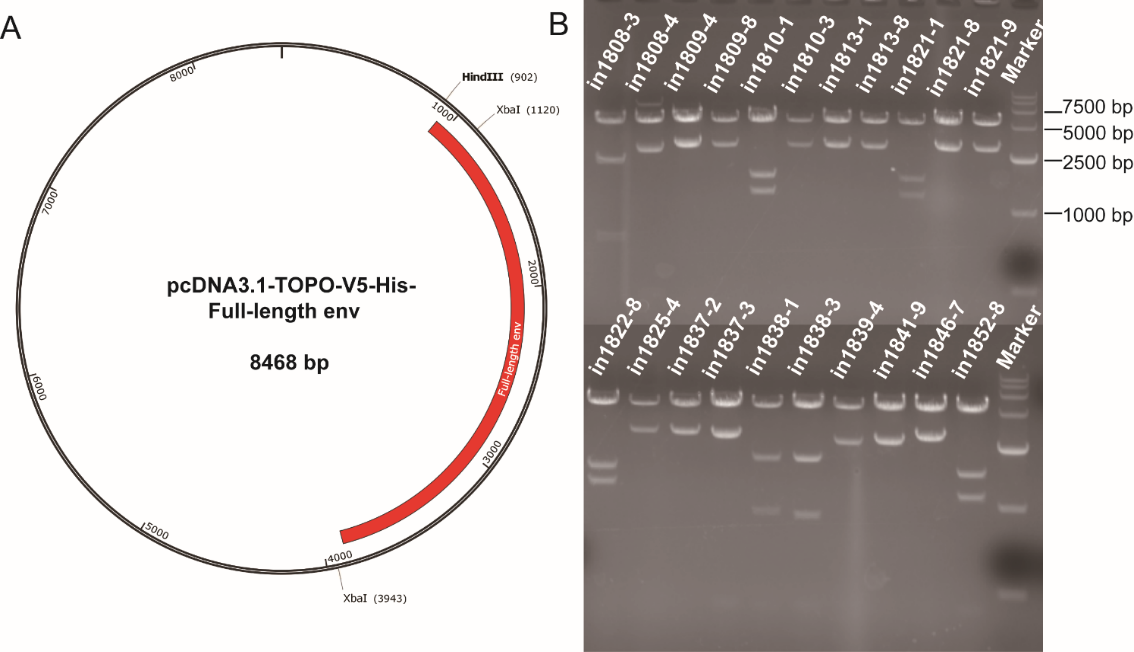


Fig.S2 Full-length *env* clone. (A) Plasmid profile of full-length *env* clone, full-length *env* gene (in red, ~3000bp) was cloned into pcDNA3.1-TOPO-V5-His vector (~5500bp). (B) Identification of full-length *env* clone by restriction endonuclease *Hin*d Ⅲ and *Xba*Ⅰ, since restriction sites may exist in the *env* gene, the result is a vector band of ~5500 bp and an *env* band of ~3000 bp (or two band with a combined size of ~3000 bp).


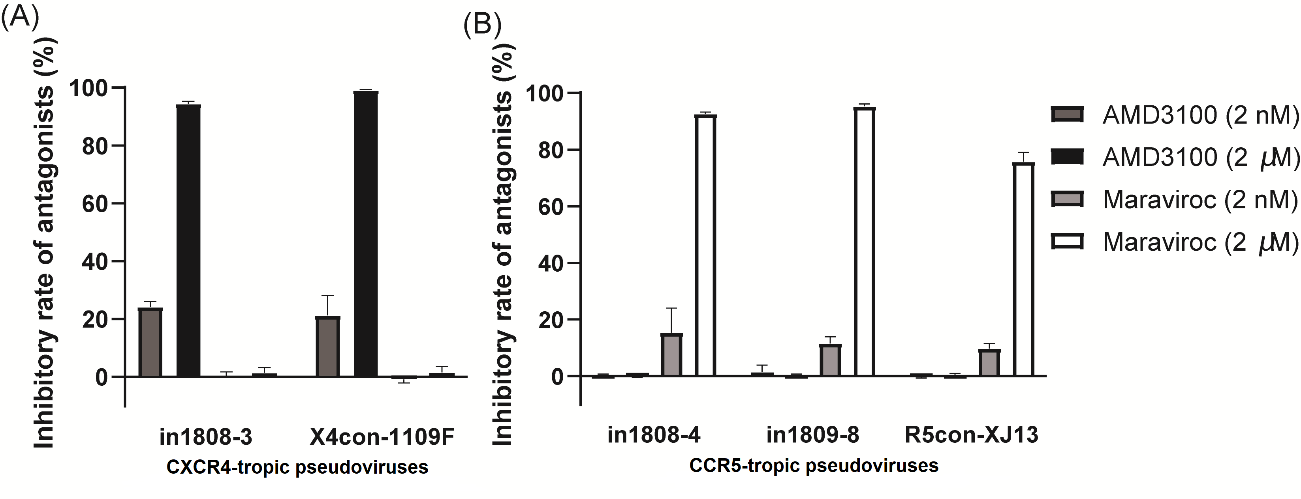


Fig.S3 Determination of tropism using coreceptor blockers. (A) Inhibitory effect of antagonists on X4 pseudoviruses. (B) Inhibitory effect of antagonists on R5 pseudoviruses. Ghost cells were treated in the absence or presence of AMD3100 (2nM or 2μM) and Maraviroc (2nM or 2μM). Approximately 15,000 cells for each sample were collected to perform flow cytometry assay for GFP signals to determine the number of infected cells. Then calculated inhibitory rate. The mean inhibitory rate is shown with standard deviations from three separate assays. X4 pseudoviruses can be blocked well by 2 µM AMD3100 and R5 pseudoviruses can be blocked well by 2 µM Maraviroc.

$$Inhibitory rate \left( \% \right)=\frac{Number of infected cells in no drugs-Number of infected cells in presence of drugs}{Number of infected cells in no drugs}\times100$$
